# Jejunal diverticulosis found in a patient with long-standing pneumoperitoneum and pseudo-obstruction on imaging: a case report

**DOI:** 10.1093/gastro/gov033

**Published:** 2015-07-27

**Authors:** Carolyn Hanna, John Mullinax, Mark S Friedman, Julian Sanchez

**Affiliations:** ^1^USF Morsani College of Medicine, Tampa, FL, USA; ^2^Department of Surgical Oncology, Moffitt Cancer Center, Tampa, FL, USA

**Keywords:** Jejunal diverticulosis, pneumoperitoneum, pseudo-obstruction

## Abstract

Small bowel diverticulosis is a rare finding within the general population and jejunal diverticulosis, specifically, is even rarer. Clinical manifestations can range from post-prandial pain, constipation and malabsorption to serious complications, such as gastro-intestinal hemorrhage, perforation and acute intestinal obstruction. Here we describe the case of an 81-year-old gentleman who presented with a three-year history of abdominal pain and weight loss. Despite unremarkable physical examination and laboratory tests, persistent pneumoperitoneum and dilated loops of small bowel were found on imaging. Having been given a diagnosis of small bowel bacterial overgrowth, the patient underwent capsule endoscopy study for further evaluation of his small bowel. The capsule did not reach the colon and the patient never noted passing the capsule in his stool so, six months post-procedure, a computed tomography (CT) scan seemed to reveal the retained capsule. Subsequent exploratory laparotomy revealed 200 cm of atonic, dilated jejunum with impressive diverticula along the anti-mesenteric border. This case report is an example of an unusual set of presenting signs and symptoms of jejunal diverticulosis, including persistent pneumoperitoneum, pseudo-obstruction and small bowel bacterial overgrowth. A literature review has revealed that these signs have been present in other cases of jejunal diverticulosis, although the etiology and pathophysiology is not clearly understood.

## Introduction

Diverticular disease is a disease of old age and of Western society [1, 2]. Uncommon under the age of 40, the prevalence of diverticulosis increases from 5% below the age of 40 to 65% in patients 65 years of age or older [1]. Diverticula are predominantly found in the left colon, with 90% involving the sigmoid colon and 15% involving right-sided colonic diverticula [3]. The incidence of diverticula of the small bowel is far less common than that of the large intestine, and within the small bowel, duodenal diverticula are five times more common than jejuno-ileal diverticula [4], the etiology of which is diverse and has been associated with progressive systemic sclerosis, visceral neuropathies and myopathies that affect the smooth muscle or myenteric plexus of the small bowel. This theoretically leads to abnormal peristalsis and increased intraluminal pressures, with the subsequent formation of diverticula [5].

In this report, we describe the case of an elderly gentleman with a history of long-standing abdominal pain, weight loss, and small bowel dilation on imaging. He was found to have impressive jejunal diverticulosis on exploratory laparotomy for what was believed to be endoscopic capsule retention.

## Case Presentation

An 81-year-old man presented for evaluation of vague abdominal pain, dyspepsia, and weight loss over the previous three years. His past medical history was significant for prostate cancer, treated more than two decades previously by prostatectomy and adjuvant radiation therapy. Prior to our evaluation, he had been diagnosed with small bowel bacterial overgrowth and was started on amoxicillin/clavulanic acid. This provided some relief of the patient’s symptoms; however he continued to have significant post-prandial pain, which led to progressive weight loss.

**Figure 1.**
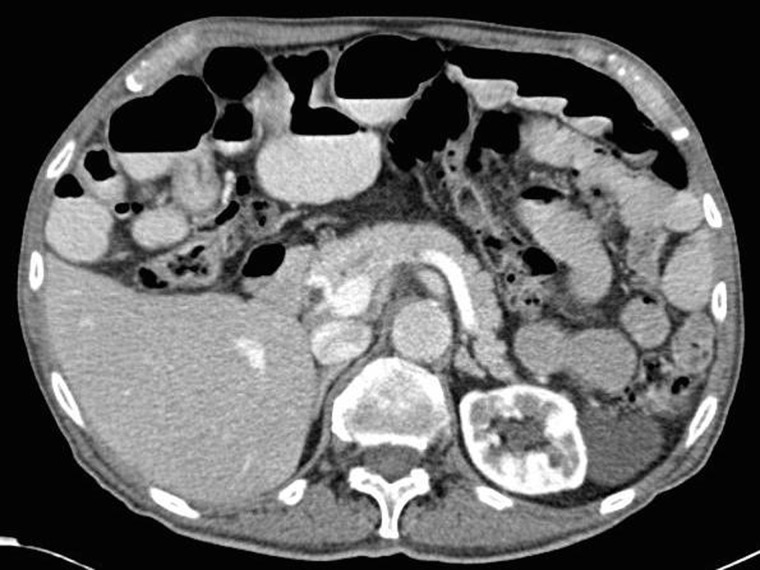
Axial CT scan of the abdomen and pelvis demonstrating extraluminal air between loops of small bowel and diffuse small bowel dilation suggestive of an obstruction.

Physical examination on his initial evaluation revealed a thin, nearly cachectic, elderly male who appeared to be his stated age. His abdominal exam was remarkable for a scaphoid abdomen, no tenderness to palpation, and a well-healed, lower midline surgical incision without hernia. Laboratory evaluation was unremarkable. Imaging included a computed tomography (CT) scan of the abdomen and pelvis with enteral and intravenous contrast. This revealed a small amount of extraluminal air between loops of small bowel and mild, diffuse small bowel dilation, suggestive of a small bowel ileus (Figure 1). The celiac, superior mesenteric and inferior mesenteric arteries were all patent at their origins, without significant ostial calcification. The patient had no symptoms consistent with ileus or small bowel obstruction. Since the extraluminal air was an incidental finding on CT and there were no signs of peritonitis on examination, surgical intervention was not recommended. Follow-up magnetic resonance imaging (MRI) of the abdomen and pelvis indicated a number of prominent small bowel loops as well, with no discrete transition point or evidence of high-grade obstruction. No evidence of small bowel tumor was found, nor focal inflammatory changes.

**Figure 2.**
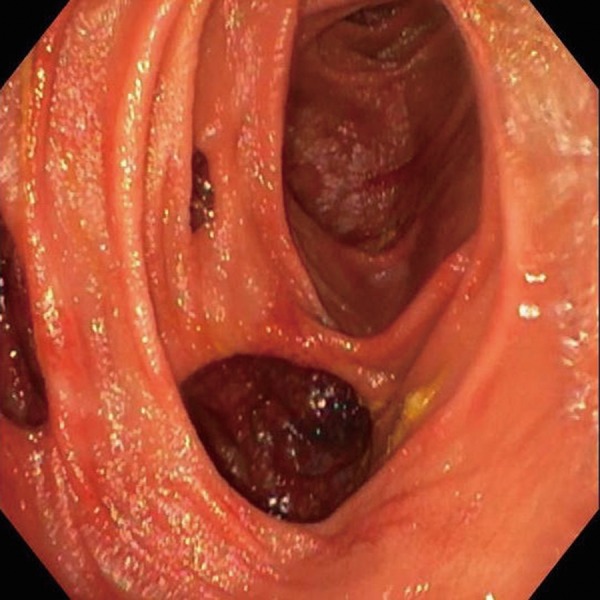
Push enteroscopy demonstrating multiple jejunal diverticula.

Colonoscopy and esophago-gastroduodenoscopy produced no adverse findings, so his evaluation proceeded with capsule endoscopy to determine the cause of the pneumoperitoneum demonstrated on the prior CT scan. This demonstrated severely inflamed proximal small bowel mucosa and what appeared to be large diverticula. The capsule had not reached the colon and the patient denied noticing that the capsule had passed in his stool. Abdominal X-ray did not reveal that any capsule was retained in either the small bowel or colon. Push enteroscopy was recommended and also demonstrated impressively large diverticulae, beginning at approximately the fourth portion of the duodenum and extending beyond the last visualized area of the proximal jejunum with significant inflammation of the jejunum (Figure 2). A water-soluble contrast fluoroscopic study of the small bowel series revealed numerous wide-mouth sacculations throughout the small bowel—most prominently within the jejunum—and overall loss of fold density within the jejunum. Diffuse smooth fold thickening was suggestive of a malabsorptive state. The patient continued to be treated for bacterial overgrowth and was also given mesalamine for the inflammation of the jejunum seen on enteroscopy.

**Figure 3.**
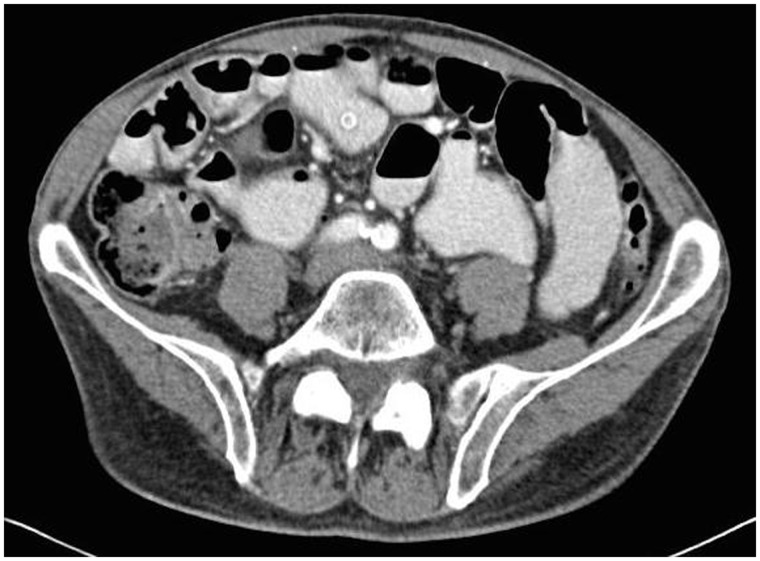
Axial CT of the abdomen and pelvis revealing what appears to be the retained capsule from a prior capsule endoscopy in the small intestine. Diffuse distension of the bowel is also present.

Approximately six months after the capsule endoscopy, the patient was seen for worsening abdominal pain, early satiety, and bloating. General Surgery service was consulted for what appeared to be visualization of the endoscopic capsule on a follow-up CT scan (Figure 3). After explaining the risks and benefits of surgical exploration, the patient consented to exploration and possible bowel resection. The decision to pursue an open—rather than a minimally invasive—approach for the operation was based on two factors: first—and most important—the degree of dilation significantly increased the risk of inadvertent enterotomy when gaining peritoneal access for a laparoscopic approach; second, the purpose of the exploration was to identify a retained endoscopic capsule and manual palpation of the bowel is required to locate the capsule; this was not possible using a minimally invasive approach.

**Figure 4.**
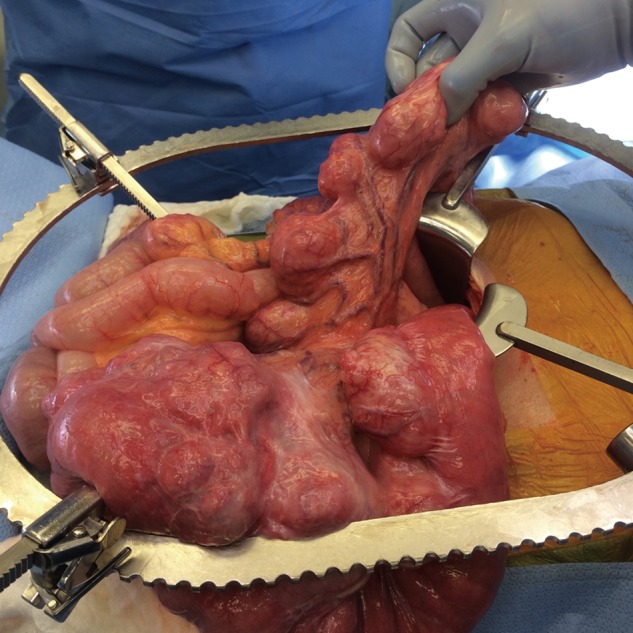
A 200 cm segment of jejunum containing large diverticula.

Several times during the exploratory laparotomy, the small bowel was thoroughly interrogated from the ligament of Treitz to the ileocecal valve, with no sign of a retained capsule. Examination of the small bowel was remarkable for diverticula extending from the ligament of Treitz about 200 cm on the antimesenteric edge of the small bowel (Figure 4). This portion of the small bowel was also very dilated and appeared atonic. Distal to this segment, the patient had 500 cm of normal-looking small bowel with appropriate caliber and thickness. After extensive exploration for vasculopathy, lymph nodes or evidence of tumor, it was elected to resect the 200 cm segment, given sufficient length of remaining small bowel. The patient tolerated the procedure well and had an uncomplicated post-operative course. He was tolerating a low-residue diet on discharge, without any post-prandial discomfort.

The patient was found to be doing well and gaining weight on follow-up approximately fifteen weeks after surgery and he denies abdominal pain, nausea or vomiting.

## Discussion

Small bowel diverticula are rare, with the prevalence at autopsies ranging from 0.6–1.5%, and imaging studies reporting an incidence of 0.002–0.7% [6, 7]. They can occur anywhere along the circumference of the bowel, but most frequently appear along—and sometimes within—the mesentery. In colonic diverticulosis, low-residue Western diets have been implicated in slower stool transit times and stool of smaller caliber, which leads to exaggerated contraction of the circular colonic muscle and increased intraluminal pressure [2, 8, 9]. Microscopic analyses of ten patients referred with jejunal diverticulosis revealed either decreased, normal-appearing muscle cells, fibrosis and degenerated smooth muscle cells, or axonal degeneration and neuronal intranuclear inclusions [5]. These findings were associated with systemic sclerosis, visceral myopathy, and visceral neuropathy, respectively; thus it is believed that these abnormalities can distort smooth muscle contraction in the small bowel and create increased intraluminal pressure, leading to mucosal and submucosal herniation at anatomic points of blood vessel (*vasa recta*) penetration [10].

Jejunal diverticulosis manifests in a variety of ways but is fortunately asymptomatic in most cases. When symptomatic, clinical manifestations of this disease may include chronic epigastric or periumbilical abdominal pain—especially post-prandial, constipation, diarrhea and malabsorption [6, 11]. In the most severe cases, the initial presentation can include life-threatening complications such as gastrointestinal hemorrhage, perforation, and acute intestinal obstruction [12]. Our patient presented with chronic, non-surgical pneumoperitoneum, pseudo-obstruction on imaging, as well as small bowel bacterial overgrowth associated with weight loss.

Recurrent pneumoperitoneum—without peritonitis secondary to jejunal diverticulosis—has been recorded in the literature only a handful of times. Wright and Lumsden described a case where a patient with jejunal diverticulosis developed spontaneous pneumoperitoneum on three separate occasions [13]. Nason and Dragan described a case where paracentesis and analysis of the aspirated gas was performed prior to laparotomy. The fluid analysis suggested that the pneumoperitoneum was of bacterial origin and from the proximal gastrointestinal tract [14]. While pneumoperitoneum is usually an ominous finding, suggestive of perforated viscous and requiring emergent surgery, few disease states can lead to pneumoperitoneum without symptoms of peritonitis and without findings of perforated viscus. In jejunal diverticulosis it is suggested that intestinal gas enters the peritoneal cavity through “minute perforations in the wall of thin-walled diverticula as a result of hyperactive peristaltic activity” [15]. Subserosal gaseous cysts have been described in histological findings from patients having undergone surgical resection of the jejunum for diverticular disease and pneumoperitoneum [16]. Surgery seems to be beneficial in resolving chronic abdominal pain and the recurrent pneumoperitoneum in these patients [15].

Mechanical obstruction can occur from volvulus or intussusception at the site of the diverticulae, or from adhesions due to diverticulitis complication [17]. Pseudo-obstruction— where no anatomical cause of obstruction is found—is reported to occur in 10–25% of patients with jejunal diverticulosis. Vomiting, abdominal distension, abdominal pain and weight loss characterize the most common presentations of chronic intestinal pseudo-obstruction. In the most severe form it can present as peritonitis from visceral perforation or incarceration of bowel. Like jejunal diverticulosis, pseudo-obstruction has also been associated with bacterial overgrowth and visceral myopathy [18]. Krishnamurthy *et al*. analysed ten patients specifically with jejunal diverticulosis and found that nine had had symptoms of intestinal pseudo-obstruction anywhere from five to forty-three years in duration. They concluded that it is a major clinical presentation of jejunal diverticulosis [5]. Another series of twenty-seven cases of pseudo-obstruction noted very similar causes of acquired jejunal diverticulosis previously outlined. Of the twenty-seven patients, the majority suffered from progressive systemic sclerosis, while the others suffered from visceral myopathy, neuropathy, sclerosing mesenteritis or jejunal diverticulosis [19].

The patient presented in this case report had been preliminarily diagnosed with small intestinal bacterial overgrowth (SIBO) and treated with oral antibiotics, resulting in some relief of his symptoms. It is important to note that some of the clinical presentations of small bowel diverticulosis discussed above, such as pseudo-obstruction and dysmotility, can contribute to the development of SIBO [20]; the bacteria in SIBO can then interfere with the metabolism of disaccharides, vitamin absorption, consumption of intraluminal protein, and deconjugation of bile acids, leading to malabsorption; thus, clinically, it can present with signs including weight loss and steatorrhea, along with abdominal pain, bloating and diarrhea. These findings are consistent with what was seen in our patient.

In conclusion, jejunal diverticulosis is an uncommon disease that is associated with dysmotility of the small intestine, leading to mucosal and submucosal herniation at points of blood vessel perforation into the intestinal wall. The presentation of the disease is clinically difficult to discern and can often lead to dire complications due to late diagnosis. One should consider such a diagnosis when no organic cause of pneumoperitoneum is found on imaging. The inability of the bowel to contract properly can also lead to pseudo-obstruction and SIBO. Surgical resection of the involved bowel is the definitive treatment in patients presenting with intractable abdominal pain—as in our patient—or for those with bleeding, perforation or mechanical obstruction [21].

## 

*Conflict of interest statement*: none declared.
